# Antihypertensive Treatment in the Elderly and Very Elderly: Always “the Lower, the Better?”

**DOI:** 10.1155/2012/590683

**Published:** 2011-09-22

**Authors:** Alberto Mazza, Emilio Ramazzina, Stefano Cuppini, Michela Armigliato, Laura Schiavon, Ciro Rossetti, Marco Marzolo, Giancarlo Santoro, Roberta Ravenni, Marco Zuin, Sara Zorzan, Domenico Rubello, Edoardo Casiglia

**Affiliations:** ^1^Department of Internal Medicine, “Santa Maria della Misericordia” Hospital, 45100 Rovigo, Italy; ^2^Department of Neuroscience, “Santa Maria della Misericordia” Hospital, 45100 Rovigo, Italy; ^3^Department of Nuclear Medicine, “Santa Maria della Misericordia” Hospital, 45100 Rovigo, Italy; ^4^Department of Clinical and Experimental Medicine, University of Padua, 35128 Padua, Italy

## Abstract

Arterial hypertension (HT) is age dependent and, with the prolongation of life expectancy, affects more and more elderly people. In the elderly, HT is a risk factor for organ damage and cardiovascular (CV) events. Both pharmacologic and nonpharmacologic reduction of blood pressure (BP) is associated with a corresponding decrease in systolic-diastolic or isolated systolic HT. Clinical trials have shown that BP lowering is associated with a decrease in stroke and other CV events. Therefore, BP reduction *per se* appears more important than a particular class of antihypertensive drugs. The benefit of antihypertensive treatment has been confirmed up to the age of 80 years, remaining unclear in the octogenarians. The benefit in lowering diastolic BP between 80 and 90 mmHg is well established, while that of lowering systolic BP below 140 mmHg requires further confirmations.

## 

The lifespan increase during the last 30 years has resulted in a remarkable raise in the world population of people aged ≥65 years [1]. Arterial hypertension (HT) is age dependent and, with the prolongation of life expectancy, affects more and more elderly people [2]. Approximately over 80% of the elderly have HT, mainly isolated systolic hypertension (ISH), defined in the European guidelines as systolic blood pressure (BP) ≥ 140 mmHg and diastolic BP < 90 mmHg [3]. ISH is an age-related condition, as systolic BP increases with advancing age, while diastolic remains unchanged or even decreases after the sixth decade of life [4]. This phenomenon produces a progressive increase in pulse pressure (PP) [5]. PP, the difference between systolic and diastolic BP, reflects the work increase due to systolic energy [6, 7]. 

In clinical practice, the decision to treat an elderly with HT depends on the answers to the following three questions

- Is HT a risk factor for stroke and cardiovascular (CV) events?
- Does non-pharmacologic and pharmacologic treatment reduce the risk of these events?
- Which is the target to achieve in the elderly hypertensives?

The aim of this paper is to answer these questions, particularly focusing the discussion on whether the paradigm “the lower, the better” maintains a prognostic role in elderly and very old hypertensives. 

In clinical trials completed before 1985, elderly hypertensive subjects were not included or represented a little component of the population under investigation [8]. At the beginning of the 90s, when the first epidemiological evidences documented the prognostic role of systolic BP [9, 10], many trials were performed in elderly hypertensives (Table 1). On the basis of the evidence provided by these trials, HT is now considered a well-established risk factor for stroke and CV disease in elderly people, and its treatment is considered as mandatory. 

In the elderly hypertensives, antihypertensive treatment is commonly recommended, but with high caution due to alterations in drug distribution and disposal, to presumptive changes in homeostatic CV control and to the quality of life that is typical of this age class. The randomized, controlled trials of antihypertensive treatment in the elderly have shown benefits comparable to those observed in younger or middle-aged subjects. Not only this, but, as the baseline CV risk is higher in the elderly, the absolute benefit of treatment (expressed as number of events prevented per 1000 patient-years) is even higher in the elderly. However, most of the hypertensives enrolled in clinical trials were <80 years old. 

The first evidence that antihypertensive treatment is also useful in subjects aged ≥80 years is that published in 1999 by Gueyffier et al., concerning a subgroup of 1,670 very old subjects taking part of the INdividual Data ANAlysis of antihypertensive intervention trials (INDANA) [11]. In this meta-analysis, antihypertensive therapy led to a reduction in stroke (−33%), CV morbidity (−22%), and heart failure (−39%). No significant effect was demonstrated for coronary events, and when the effect of treatment on fatal and nonfatal stroke was analyzed separately the benefit was limited to the nonfatal only. Ten years later, similar results were partially confirmed in the Hypertension in the Very Elderly Trial (HYVET) over 3,845 subjects aged ≥80 years and having high systolic BP [12], where all subjects were randomly assigned to placebo or active treatment with indapamide and perindopril was added in individuals who failed to meet the target BP of 150/80 mmHg. At two years of followup, mean BP was 15/6 mmHg lower in subjects receiving active treatment than in those receiving the placebo, a difference that was associated with significant reduction of death from stroke, both fatal and non-fatal (−30%), cardiovascular disease (−23%), and heart failure (−64%). 

In the HYVET, a 21% reduction of the risk of overall mortality was also observed with active treatment. Nevertheless, the results of Bejan-Angoulvant's meta-analysis did not support those of the HYVET, showing comparable overall mortality in treated and untreated patients [13]. This discrepancy was outlined in the recent joint consensus developed by the American College of Cardiology Foundation and the American Heart Association [14]. The subjects enrolled in the HYVET were in good physical and mental condition and had low rate of previous CVD and therefore were not representative of very elderly. 

Systolic HT (≥140 mmHg) and pulse HT (≥80 mmHg) [6] characterise the pressure profile of elderly hypertensives. It is therefore only natural that the intervention trials were focused on reducing systolic BP. The current ESH/ESC guidelines recommend reducing systolic BP below 140 mmHg in grade 1-2 hypertensives having low-to-moderate total CV risk. Nevertheless, whether this recommendation also applies to elderly and very old subjects is unproved by outcome trials. In all trials [15–22] but one [23], elderly hypertensives randomized to more active treatment had lower incidence of CV events, but in no trial the systolic target (<140 mmHg) was reached. The ACCOMPLISH [24] and the INVEST [25] studies showed no difference in antihypertensive effects when comparing drug treatment in subjects of age ≥80 or <80 years, implicitly supporting the opportunity to treat very old subjects. Nevertheless, the Japanese Trial to Assess Optimal Systolic (JATOS) blood pressure in elderly hypertensive patients over-65–85-year-old subjects, (JATOS) demonstrated that a more strict BP control did not provide further benefit in reducing stroke, heart disease, vascular disease, and renal failure [23] and even showed a negative result on CV events suggesting a possible deleterious effect of intensive BP control in elderly hypertensives. This is not peculiar of old subjects, being in agreement with the results of the ACCORD trial [26] that showed no additional benefit of BP reduction—but only an increase in drug-related adverse effects in—high-risk patients with diabetes mellitus ≥55 years when targeting systolic at 120 rather than 140 mmHg. In addition, observational data from INVEST in hypertensive patients with coronary artery disease showed a J-curve pattern for adverse outcomes at on-treatment systolic BP of 135 mmHg in patients aged 70 to 79 years and at 140 mmHg for those aged ≥80 years. This is not a new notation, as some retrospective analyses of intervention studies suggested [27] with exceptions [17–22] a J-curve trend of the risk of myocardial infarction in relation to treated BP. Also in a posthoc analysis of the EWPHE [15] it appears that in elderly hypertensives under active treatment total mortality had a U-shaped trend in relation to systolic BP, with a nadir about 150 mmHg, whereas total mortality increased gradually with decreasing DBP from the upper tertile of 98 mmHg (these results were partially flawed by the fact that a U-shaped trend with a nadir at 95 mmHg was also found in the patients taking placebo, so that conclusive inferences cannot be drawn from this retrospective analysis). Finally, in the Hypertension Optimal Treatment (HOT) study [28], where 30% of the hypertensives were older than 65 years, it was found that the optimal BP for the lowest incidence of CV events was 138 mmHg for systolic and 83 mmHg for diastolic, with no significant improvement in CV end-points when BP was led to lower levels. The intention-to-treat analysis revealed a comparable pattern in the incidence of CV events in the adults and in the older patients, suggesting that optimal BP reductions are similar and independent of age.

Therefore, no trial evidence supports the guidelines recommendation to achieve a systolic target <140 mmHg in elderly subjects; in particular systolic values <130 and diastolic <65 mmHg should probably be avoided in the elderly. 

In conclusion, particular attention should be paid to antihypertensive treatment of elderly hypertensives, which constitute a large, growing, and vulnerable part of general population. There is no doubt that antihypertensive treatment is justified by medical evidence. The assumption “the lower systolic BP, the lower the risk” is adequate for stroke and heart failure. Despite this, the best meta-analysis showed no clear results in decreasing total mortality by forcing antihypertensive treatment in very old subjects. In the randomized-controlled trials, elderly hypertensives were treated with diuretics, *β*-blockers, dihydropyridines calcium channel blockers, and converting-enzyme inhibitors. However, monotherapy normalizes BP in only 40–50% of cases, and therefore a combination of two or more drugs is often required to achieve the recommended BP goals. The most reasonable strategy is to start with a thiazide diuretic as first-line therapy and to optimize the maximal antihypertensive therapy with two drugs in low doses. The JNC, the WHO/ISH, and ESH/ESC guidelines recommend lowering BP in elderly hypertensives below 140/90 mmHg. In this respect there are sufficient data that a diastolic BP between 80 and 90 mmHg is associated with a clear benefit, except in case of coronary heart disease where a mortality increase was observed reducing diastolic BP below 80 mmHg.

## Figures and Tables

**Table 1:** Efficacy of the antihypertensive treatment in stroke and cardiovascular events in different trials performed in the elderly.

Trial	Mean age at randomization (years)	Subjects enrolled	Mean BP at randomization (mmHg)		Drug treatment	Mean followup (years)	Stroke Reduction (%)	CV events reduction (%)
			SBP	DBP				
Coope/Warrender	68	884	196	99	Atenolol; Bendrofluazide	4,04	−30	—
EWPHE	72	840	183	101	HCTZ; Triamterene; Methyldopa	8	NS	− 27
HYVET	84	3845	173	91	Indapamide; Perindopril	2	−30	−34
MRC-HT	70	4396	185	91	Atenolol; HCTZ; Amiloride	5,8	−31	−35
SHEP	72	4716	170	77	Chlorthalidone	4,5	−36	−32
STONE	67	1632	180	90	Nifedipine (Long-acting)	2,5	−57	−60
STOP-HTN	76	1627	195	94	Atenolol; HCTZ; Amiloride; Metoprolol; Pindolol	5	−47	−40
Syst-China	67	3000	171	86	Nitrendipine; Captopril; HCTZ	2	−38	−37
Syst-Eur	70	4695	174	85	Nitrendipine; Enalapril; HCTZ	2	−42	−31

SBP: systolic blood pressure; DBP: diastolic blood pressure; EWPHE: European Working Party on High blood pressure in the Elderly trial; HYVET: hypertension in the very elderly; MCR: *Medical Research Council Hypertension Trial;* SHEP: systolic hypertension in the elderly; STONE: Shanghai Trial of Hypertension in the Elderly; STOP-HTN: Swedish Trial in Old Patients with Hypertension; Syst-China: systolic hypertension in China; Syst-Eur: Systolic Hypertension in Europe; NS: not significant; HTCZ: hydrochlorothiazide.
